# Transcranial Magnetic Stimulation for Status Epilepticus

**DOI:** 10.1155/2015/678074

**Published:** 2015-11-22

**Authors:** F. A. Zeiler, M. Matuszczak, J. Teitelbaum, L. M. Gillman, C. J. Kazina

**Affiliations:** ^1^Section of Neurosurgery, Department of Surgery, University of Manitoba, Winnipeg, MB, Canada R3A 1R9; ^2^Undergraduate Medicine, University of Manitoba, Winnipeg, MB, Canada R3A 1R9; ^3^Section of Neurology, Montreal Neurological Institute, McGill, Montreal, QC, Canada H3A 2B4; ^4^Section of Critical Care Medicine, Department of Medicine, University of Manitoba, Winnipeg, MB, Canada R3A 1R9; ^5^Section of General Surgery, Department of Surgery, University of Manitoba, Winnipeg, MB, Canada R3A 1R9

## Abstract

*Background*. Our goal was to perform a systematic review on the use of repetitive transcranial magnetic stimulation (rTMS) in the treatment of status epilepticus (SE) and refractory status epilepticus (RSE).* Methods*. MEDLINE, BIOSIS, EMBASE, Global Health, Healthstar, Scopus, Cochrane Library, the International Clinical Trials Registry Platform, clinicaltrials.gov (inception to August 2015), and gray literature were searched. The strength of evidence was adjudicated using Oxford and GRADE methodology.* Results*. We identified 11 original articles. Twenty-one patients were described, with 13 adult and 8 pediatric. All studies were retrospective. Seizure reduction/control with rTMS occurred in 15 of the 21 patients (71.4%), with 5 (23.8%) and 10 (47.6%) displaying partial and complete responses, respectively. Seizures recurred after rTMS in 73.3% of the patients who had initially responded. All studies were an Oxford level 4, GRADE D level of evidence.* Conclusions*. Oxford level 4, GRADE D evidence exists to suggest a potential impact on seizure control with the use of rTMS for FSE and FRSE, though durability of the therapy is short-lived. Routine use of rTMS in this context cannot be recommended at this time. Further prospective study of this intervention is warranted.

## 1. Introduction

Repetitive transcranial magnetic stimulation (rTMS) has recently been employed as a treatment option for psychiatric conditions [1], chronic pain [2], movement disorders [3], and epilepsy [4, 5]. The use of rTMS for the control of medically refractory epilepsy has increased in the last 15 years, with over 30 publications since 1990 [5].

The exact mechanism of action of rTMS in seizure control is unknown. It is proposed that the long term effects in terms of seizure reduction are related to a reduction in cortical excitability secondary to long term depression or potentiation [5], with long term depression/potentiation referring to a use-dependent modulation of synaptic strength.

Animal kindling models in epilepsy have displayed the antiepileptic effect of rTMS [6, 7], with a potential frequency dependent impact on seizure control [7, 8]. In humans, a recent systematic review of rTMS for refractory epilepsy has displayed the safety and tolerability with improvement in seizure frequency in the majority of studies [5]. Furthermore, recent arguments have surfaced supporting the cost effectiveness of rTMS for refractory epilepsy over standard failed antiepileptic drug (AED) based therapies [9]. Overall, recent evidence based guidelines support level C evidence for rTMS in the treatment of epilepsy [10].

Status epilepticus (SE) and refractory status epilepticus (RSE) pose difficult therapeutic challenges. Novel therapies such as rTMS have been sought out to treat RSE cases [10, 11], with a small number of cases reported in the literature to date [12–23]. The efficacy of rTMS in the setting of SE and RSE is currently unclear.

Our goal was to perform a systematic review of the literature on the use of rTMS for the treatment of SE and RSE.

## 2. Materials and Methods

A systematic review using the methodology outlined in the Cochrane Handbook for Systematic Reviewers [24] was conducted. The data was reported following the Preferred Reporting Items for Systematic Reviews and Meta-Analyses (PRISMA) [25]. The review questions and search strategy were decided upon by the primary author (F. A. Zeiler) and supervisor (C. J. Kazina).

### 2.1. Search Question, Population, and Inclusion and Exclusion Criteria

The question posed for systematic review was the following: What is the effectiveness of rTMS in the treatment of SE/RSE? We utilized the Neurocritical Care Society guidelines on the management of SE based definition of SE and RSE [26]. The term generalized refractory status epilepticus (GRSE) was used to refer to generalized RSE. The term focal refractory status epilepticus (FRSE) was used to refer focal RSE. The term multifocal refractory status epilepticus (MFRSE) was used to refer to RSE that had a multifocal nature. The term nonconvulsive refractory status epilepticus (NCRSE) was used for nonconvulsive seizures that fulfilled the criteria for RSE.

All studies, prospective and retrospective of any size based on human subjects, were included. The reason for an all-inclusive search was based on the small number of studies of any type identified by the primary author during a preliminary search of MEDLINE and EMBASE.

The primary outcome measure was electrographic seizure control, defined as complete resolution, partial seizure reduction, and failure. Secondary outcome measures were patient outcome (if reported), and adverse effects to rTMS.

Inclusion criteria were as follows: all studies including human subjects whether prospective or retrospective, all study sizes, any age category, and the documented use of rTMS treatment for the purpose of seizure control in the setting of SE/RSE. Exclusion criteria were as follows: animal and non-English studies.

### 2.2. Search Strategy

MEDLINE, BIOSIS, EMBASE, Global Health, Healthstar, SCOPUS, and Cochrane Library from inception to August 2015 were searched using individualized search strategies for each database. The search strategy for MEDLINE can be seen in Appendix  A of the Supplementary Material available online at http://dx.doi.org/10.1155/2015/678074, with a similar search strategy utilized for the other databases. In addition, the World Health Organizations International Clinical Trials Registry Platform and ClinicalTrials.gov were searched looking for studies planned or underway, with none identified.

Additionally, meeting proceedings for the last 10 years looking for ongoing and unpublished work based on TMS for SE/RSE were examined. The meeting proceedings of the following professional societies were searched: Canadian Neurological Sciences Federation (CNSF), American Association of Neurological Surgeons (AANS), Congress of Neurological Surgeons (CNS), European Neurosurgical Society (ENSS), World Federation of Neurological Surgeons (WFNS), American Neurology Association (ANA), American Academy of Neurology (AAN), European Federation of Neurological Science (EFNS), World Congress of Neurology (WCN), Society of Critical Care Medicine (SCCM), Neurocritical Care Society (NCS), World Federation of Societies of Intensive and Critical Care Medicine (WFSICCM), American Society for Anesthesiologists (ASA), World Federation of Societies of Anesthesiologist (WFSA), Australian Society of Anesthesiologists, International Anesthesia Research Society (IARS), Society of Neurosurgical Anesthesiology and Critical Care (SNACC), Society for Neuroscience in Anesthesiology and Critical Care, and the Japanese Society of Neuroanesthesia and Critical Care (JSNCC).

Finally, reference lists of any review articles or systematic reviews on seizure management were reviewed for relevant studies on the use of rTMS for the treatment of SE/RSE that were missed during the database and meeting proceeding search.

### 2.3. Study Selection

Utilizing two reviewers (F. A. Zeiler and M. Matuszczak), a two-step review of all articles returned by our search strategies was performed. First, the reviewers independently screened all titles and abstracts of the returned articles to decide if they met the inclusion criteria. Second, full text of the chosen articles was then assessed to confirm if they met the inclusion criteria and that the primary outcome of seizure control was reported in the study. Any discrepancies between the two reviewers were resolved by a third party (C. J. Kazina).

### 2.4. Data Collection

Data was extracted from the selected articles and stored in an electronic database. Data fields included patient demographics, type of study (prospective or retrospective), number of patients, rTMS coil used, timing to rTMS treatment, rTMS treatment parameters, time to effect of rTMS, how many other AEDs were utilized prior to implementation of rTMS, degree of seizure control (as described previously), adverse effects to rTMS, and patient outcome (if recorded).

### 2.5. Quality of Evidence Assessment

Assessment of the level of evidence for each included study was conducted by a panel of two independent reviewers, utilizing the Oxford criteria [27] and the Grading of Recommendation Assessment Development and Education (GRADE) criteria [28–33] for level of evidence. We elected to utilize two different systems to grade level of evidence given that these two systems are amongst the most commonly used. We believe this would allow a larger audience to follow our systematic approach in the setting of unfamiliarity with a particular grading system.

The Oxford criteria consists of a 5-level grading system for literature. Level 1 is split into subcategories 1a, 1b, and 1c which represent a systematic review of randomized control trials (RCT) with homogeneity, individual RCT with narrow confidence interval, and all or none studies, respectively. Oxford level 2 is split into 2a, 2b, and 2c representing systematic review of cohort studies with homogeneity of data, individual cohort study or low quality RCT, and outcomes of research, respectively. Oxford level 3 is split into 3a and 3b representing systematic review of case-control studies with homogeneity of data and individual case-control study, respectively. Oxford level 4 represents case series and poor cohort studies. Finally, Oxford level 5 represents expert opinion.

The GRADE level of evidence is split into 4 levels: A, B, C, and D. GRADE level A represents high evidence with multiple high quality studies having consistent results. GRADE level B represents moderate evidence with one high quality study, or multiple low quality studies. GRADE level C evidence represents low evidence with one or more studies with severe limitations. Finally, GRADE level D represents very low evidence based on either expert opinion or few studies with severe limitations.

Any discrepancies between the grading of the two reviewers (F. A. Zeiler and M. Matuszczak) were resolved via a third party (C. J. Kazina).

### 2.6. Statistical Analysis

A meta-analysis was not performed in this study due to the heterogeneity of data within the articles and the presence of a small number of low quality retrospective studies.

## 3. Results

**Figure 1:**
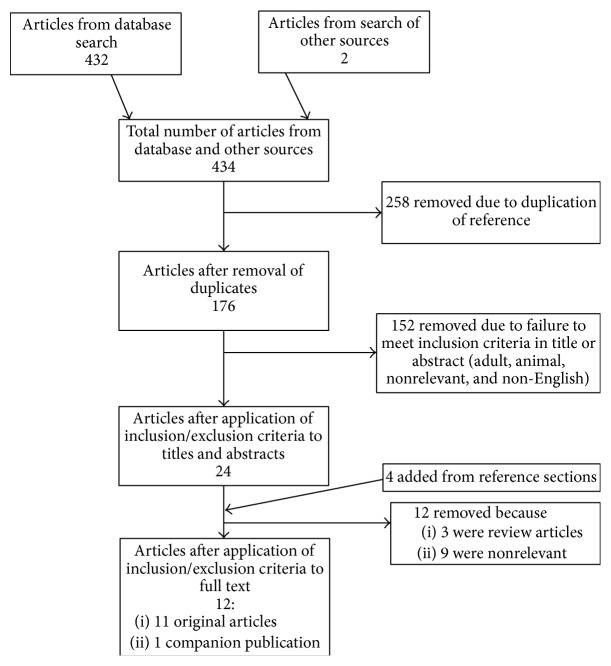
Flow diagram of search results.

The results of the search strategy across all databases and other sources are summarized in Figure 1. Overall a total of 434 articles were identified, with 432 from the database search and 2 from the search of published meeting proceedings. After removing duplicates, there were 176 articles. By applying the inclusion/exclusion criteria to the title and abstract, we identified 24 articles that fit these criteria with 22 from the database search and 2 from published meeting proceedings. Applying the inclusion/exclusion criteria to the full text documents, only 8 articles were eligible for inclusion, with 6 from database and 2 from meeting proceedings. The other articles were excluded because they either did not report details around the use of rTMS for seizure control, or because they were review articles. Reference sections from review articles were searched for any other articles missed in the database search, with 4 being identified. These were subsequently added to make a total of 12 articles for the final review.

Of the 12 articles included in the review [12–23], 11 were original studies [12–22] and 1 was a companion publication [23] with duplicate patient data. Rotenberg et al. [23] was a case report of Rasmussen's encephalitis treated with rTMS, which was subsequently also reported in the case series of rTMS for FSE, Rotenberg et al. [18]. In order to avoid duplication of patient data, Rotenberg et al. [23] was not included in the final data summary.

All 11 original studies were retrospective studies [12–22], with 5 retrospective case series [12, 14, 16, 18, 20] and 6 retrospective case reports [13, 15, 17, 19, 21, 22]. All were single center reports. Six studies described the use of rTMS for SE/RSE in adult patients only [14, 15, 17, 19, 21, 22]. Four studies described the use of rTMS in pediatric patients only [12, 13, 16, 20]. One study described the use of rTMS in both adult and pediatric patients [18].

Across all studies, a total of 21 patients were documented as having being treated with rTMS for SE/RSE (mean: 1.9 patients/study; range: 1–7 patients/study). Eight pediatric patients were treated, with a mean age of 8.3 years (age range: 2.66 years to 16 years). Thirteen patients were adult with a mean age of 42.3 years (age range: 18 to 79 years).

Seizures were classified as FSE in 10 patients [15, 18, 20], GRSE in 2 patients [14, 17], FRSE in 8 patients [12, 14, 16, 19, 21, 22], and nondefined SE/RSE in 1 patients [13].

The etiology of SE/RSE varied significantly and was as follows: primary epilepsy in 5 patients [12, 14, 21], stroke in 2 patients [16, 18], hypoglycemia in 2 patients [18], Rasmussen's encephalitis in 2 patients [18, 22], Dravet syndrome in 1 patient [13], focal cortical dysplasia in 1 patient [15], lipofuscinosis in 1 patient [16], postanoxic brain injury in 1 patient [17], post vascular malformation resection in 1 patient [18], herpes simplex encephalitis in 1 patient [19], Alpert's disease in 1 patient [20], nondefined “cortical malformation” in 1 patient [20], and unknown in 2 patients [18].

Study demographics and patient characteristics for all studies can be seen in Table 1, while treatment characteristics and seizure outcome are reported in Table 2.

### 3.1. rTMS Treatment Characteristics

Nine of the 11 original articles provided [12, 14–21] details around the treatment parameters for rTMS. The 2 remaining articles referred to the use of rTMS in the management of SE/RSE, without providing any further information [13, 22].

Fourteen patients were treated with a figure 8 coil configuration [12, 14–16, 18, 19]. Two patients were treated with a “round” coil [16, 17]. Finally, 5 patients were treated with a nonspecified coil type [13, 20–22]. The stimulation parameters were highly heterogeneous between the patients described. The number of trains applied varied from 1 to 15. The frequency of stimulation varied from 0.5 Hz to 20 Hz. The train duration varied from 2 to 1800 seconds. The intertrain delay was poorly documented. Many patients received different treatment regimens on separate days [18].

The duration of rTMS treatment for these studies also varied dramatically. Some studies described a single treatment [14, 18], while others described 2 or more (range: 2 consecutive days up to 2 weeks) treatment sessions with the most aggressive schedule describing an 8-day course with varying once or twice per day stimulation settings [19].

Duration of treatment prior to the use of rTMS was documented in 3 articles [14, 17, 19], ranging from 7 to 44 days (mean = 22.0 days). The remaining 8 articles failed to mention the duration of therapy prior to rTMS. The number of AEDs administered prior to TMS was variable and was documented in 8 studies [12, 14–17, 19, 21, 22], with the total number ranging from 1 to 15 (mean = 7.5, median = 7.5).

Treatment characteristics for the adult studies can be seen in Table 2.

### 3.2. Seizure Response

Seizure response to rTMS in the setting of SE/RSE occurred in 15 of the 21 patients (71.4%) included in the review, with 5 patients [14, 15, 20] (23.8%) displaying partial EEG based response and 10 patients [12, 13, 17–19, 21] (47.6%) displaying complete resolution of seizures. Six patients (28.6%) had no response to rTMS [12, 16, 18, 22]. The time to seizure response with rTMS was documented in only 2 studies [12, 18] with response occurring either during treatment [18] or following therapy up to 24 hours [12].

Looking at seizure subtype: 8 of the 10 (80.0%) FSE patients responded, 4 of the 8 (50.0%) FRSE patients responded, the 2 GRSE patients responded (100%), and the 1 “unknown” SE/RSE patient (100%) responded to TMS.

Seizure recurrence occurred in 11 of the 15 patients (73.3%) who initially responded. The time frame to seizure recurrence was quite variable, ranging from 72 hours up to 4 months. The duration of response was not documented in 5 patients in whom a response to rTMS was noted [13, 16, 20, 21].

### 3.3. Adverse Effects of rTMS

Nine studies documented the presence or absence of adverse events related to rTMS [12, 14–21]. Two studies failed to mention any assessment for adverse events [13, 22]. Only 1 patient was described as having an adverse event secondary to rTMS. This patient developed transient leg sensory problems which completely resolved [16].

### 3.4. Outcome

Outcome data was poorly recorded in the majority of the studies included within the review. Data on patient outcome longer than 6 months was unavailable in all studies included in the review. The majority of rTMS responders had recurrence of seizures at variable time frames after treatment, as described above. This led to either repeated treatment with rTMS, or other interventions such as operative disconnection procedures or vagal nerve stimulators. Outcomes are summarized in Table 2.

No identifiable trend in outcomes could be seen based on seizure subtype or etiology of seizure.

### 3.5. Level of Evidence for rTMS

Based on the 11 original articles included in the final review, all fulfill Oxford level 4, GRADE D evidence to suggest some potential impact of rTMS on seizure control for FSE and FRSE. The role of rTMS for GRSE is unclear given the limited data.

Summary of the level of evidence can be seen in Table 3.

## 4. Discussion

We decided to perform an extensive systemic review of the literature in order to determine the effect of rTMS in the setting of SE/RSE. During the review we identified 11 original articles [12–22]. Twenty-one patients were described within these articles, with 13 being adult and 8 being pediatric. For the 8 pediatric patients who were treated, the mean age was 8.3 years (age range: 2.66 years to 16 years). For the 13 adult patients the mean age was 42.3 years (age range: 18 to 79 years). All studies were retrospective in nature. Seizure reduction/control with rTMS occurred in 15 of the 21 patients (71.4%), with 5 (23.8%) and 10 (47.6%) displaying partial and complete responses, respectively. Seizures recurred after rTMS in 73.3% of the patients who had initially responded. One patient had a transient adverse event after rTMS which completely resolved. Patient outcome data was too sparingly documented for any strong conclusion, with no identifiable trend in outcomes for the responders versus the nonresponders, or based on seizure subtype or etiology. All studies were an Oxford level 4, GRADE D level of evidence. Thus, based on this review, we can currently provide Oxford level 4, GRADE D recommendations that rTMS may provide some impact on seizure control in the setting of FSE and FRSE.

A few important points can be seen within our review. First, rTMS seems quite effective for FSE with an 80% overall response rate. Second, rTMS for FRSE has a moderate efficacy of 50% compared to the results in FSE. This highlights the ongoing resistance to therapies seen with progressive and uncontrolled seizures. Furthermore, it suggests that the role for rTMS in FSE/FRSE is earlier rather than later in the treatment algorithm. Further prospective analysis of rTMS for this indication needs to occur. Third, we are unfortunately unable to comment on the efficacy of rTMS for GSE/GRSE given the limited cases described to date. Fourth, the treatment durability of rTMS is limited, with recurrence of seizures occurring within 72 hours up to 4 months in 73.3% of initial responders. This highlights that rTMS for FSE/FRSE is a technique for potentially rapid and acute control, thus acting as a transition therapy to an altered oral AED regimen or future regular rTMS treatment protocol. Fifth, the optimal rTMS stimulation parameters that lead to seizure control/reduction in SE/RSE are not well defined and, based on this review, remain currently unclear. Finally, there were a small number of complications described within the literature included in the review. This appears to mirror the data available for other pathologies treated with rTMS [1–5].

Despite the interesting results, our systematic review has significant limitations. First, the small number of studies identified, all with small patient populations, makes it difficult to generalize to all SE/RSE patients. Furthermore, our comments on the impact of rTMS for SE/RSE are currently limited to FSE/FRSE given the limited data for other subsets refractory seizures. Second, we were unable to perform a meta-analysis given the retrospective heterogeneous nature of the data. Third, as acknowledged previously, the optimal rTMS stimulation parameters which lead to seizure response in SE/RSE are unclear. The heterogenous treatment plans for the patients identified in the review produce a confusing picture on optimal stimulation strategy. Further prospective studies will need to be conducted in order to determine efficacy and treatment regimens. Fourth, the seizure response to rTMS may not be related entirely to the stimulation alone, and may represent a reflection of the combination of multiple AEDs. Fifth, and probably most importantly, there is a potential for publication bias in the articles reviewed. We suspect that publication bias is quite high in the literature identified. It is likely that there are many more failed cases of rTMS for SE/RSE that have not been published. Finally, comments related to patient outcomes are limited, and the impact of rTMS on long term patient outcome cannot be made at this time.

Routine use of rTMS for SE/RSE cannot be recommended at this time. The results of this review point to a potential impact rTMS may have on seizure control in FSE/FRSE. Further prospective study is clearly warranted in order to better define the role of rTMS in the context of SE/RSE. International databases for SE/RSE patients with prospectively maintained data could potentially bolster the data set for rTMS, and other salvage therapies for refractory seizures.

## 5. Conclusions

Oxford level 4, GRADE D evidence exists to suggest a potential impact on seizure control with the use of rTMS for FSE and FRSE, though durability of the therapy is short-lived. Routine use of rTMS in this context cannot be recommended at this time. Further prospective study of this intervention is warranted in order to determine its true efficacy in FSE/FRSE, amongst other subtypes of SE and RSE.

## Supplementary Material

Appendix A displays a sequential listing of the exact search terms and operators utilized in our MEDLINE database search. This search strategy was similar for all of the databases listed.

## Figures and Tables

**Table 1:** Adult study characteristics and patient demographics.

Reference	Number of patients treated with rTMS	Study type/design	Article location	Mean age (years)	Etiology of seizures and type of SE/RSE	Mean # AED prior to rTMS	Mean time until rTMS administration (days)
Graff-Guerrero et al. [12]	2	Retrospective case series	Journal manuscript	9 (11 and 7 yrs)	*Etiology*: primary epilepsy (2) *Type*: FRSE	4	N/A

Hyllienmark and Åmark [13]	1	Retrospective case report	Journal manuscript	5	*Etiology:* Dravet syndrome *Type*: cryptogenic SE	N/A	N/A

Liu et al. [14]	2	Retrospective case series	Journal manuscript	49 (46 and 51 yrs)	*Etiology*: primary epilepsy (2) *Type*: 1 → GRSE 2 → FRSE	8	15

Misawa et al. [15]	1	Retrospective case report	Journal manuscript	31	*Etiology: *FCD *Type*: FSE	1	N/A

Morales et al. [16]	2	Retrospective case series	Journal manuscript	12 (8 and 16 yrs)	*Etiology: *lipofuscinosis (1) and congenital infarct (1) *Type*: FRSE	4	N/A

Naro et al. [17]	1	Retrospective case report	Journal manuscript	35	*Etiology:* Postanoxic brain injury *Type*: GRSE	3	7

Rotenberg et al. [18]	7	Retrospective case series	Journal manuscript	41 (range: 11 to 79 yrs)	*Etiology: *hypoglycemia (2); postvascular malformation resection (1); stroke (1); Rasmussen's encephalitis (1); unknown (2) *Type*: FSE	N/A	N/A

Thordstein and Constantinescu [19]	1	Retrospective case report	Journal manuscript	68	*Etiology: *HSV encephalitis *Type*: FRSE	8	44

Thordstein et al. [20]	2	Retrospective case series	Meeting abstract	4.5 (2 yrs, 8 mons and 6 yrs, 3 mons)	*Etiology: *Alpert's (1) and cortical malformations (1) *Type*: 1 → FSE 2 → FSE	N/A	N/A

Van Haerents et al. [21]	1	Retrospective case report	Meeting abstract	24	*Etiology:* primary epilepsy *Type*: FRSE	7	N/A

Wusthoff et al. [22]	1	Retrospective case report	Journal manuscript	29	*Etiology: *Rasmussen's encephalitis *Type*: FRSE	15	N/A

Rotenberg et al. [23]	1	Retrospective case report	Journal manuscript	14	*Etiology*: Rasmussen's encephalitis *Type*: FRSE	8	N/A

rTMS: repetitive transcranial magnetic stimulation; AED: antiepileptic drug; N/A: not available; SE: status epilepticus; FSE: focal status epilepticus; FRSE: focal refractory status epilepticus; GRSE: generalized refractory status epilepticus; yrs: years; mons: months; FCD: focal cortical dysplasia; HSV: herpes simplex virus. Rotenberg et al. [18] contains a series of patients including the case description from Rotenberg et al. [23]. Thus, the data from Rotenberg et al. [23] was not included in the final summary and analysis of data in order to avoid duplication of patient data.

**Table 2:** rTMS treatment characteristics, seizure response, and outcome.

Reference	Number of patients treated with rTMS	rTMS coil type	rTMS treatment regimen (trains/freq./ train duration)	Other AEDs on board	Electrographic seizure response	Duration of response	Adverse effects to rTMS	Patient outcome
Graff-Guerrero et al. [12]	2	Figure of 8	15/20 Hz/2 s train with intertrain of 58 s	1→ Valproic acid Phenytoin Primidone Topiramate 2→ Phenytoin Clobazam Valproic acid Oxcarbazepine	1→ seizure cessation after 24 h 2→ slight frequency decrease in epileptic spikes	1→ 2 weeks 2→ N/A	None	1→ required hemispherectomy: biopsy showed Rasmussen's encephalitis 2→ minimal improvement

Hyllienmark and Åmark [13]	1	N/A	N/A	Lidocaine Midazolam Thiopental	Burst suppression	N/A	N/A	Good, seizures ceased

Liu et al. [14]	2	Figure of 8	1→ 1/1 Hz/1200 s 2→ 1/1 Hz/1800 s	1→ Phenobarbital Pregabalin Lamotrigine Fosphenytoin Lacosamide Levetiracetam Pentobarbital 2→ Lamotrigine Levetiracetam Felbatol Lorazepam Lacosamide	1→ seizure frequency and spike detections decreased 2→ seizure frequency decreased	1→ until discharge (4 weeks) 2→ 72 hours	None	1→ discharged and sent to rehab on day 47 2→ required further vagus nerve stimulation; returned to baseline and discharged on 11 days after TMS

Misawa et al. [15]	1	Figure of 8	100 pulses at 0.5 Hz	Clonazepam Phenytoin	FSE suppression in hand but FSE in foot persisted	3 months	None	Patient underwent second TMS treatment which resulted in FSE suppression for 2 months

Morales et al. [16]	2	1→ round coil (5 cm diameter) 2→ figure of 8	1→ 2 sessions: 4/1 Hz/600 s and 10/6 Hz/5 s trains with 25-second intertrain interval followed by 1/1 Hz/600 s 2→ 2 sessions: 1/1 Hz/900 s and 10/6 Hz/5 s with 25 s intertrain interval followed by 1/1 Hz/900 s	1→ Zonisamide Phenobarbital Coenzyme Q Levetiracetam Carnitine 2→ Lamotrigine Clobazam	1→ no response 2→ no response	N/A	1 none 2 increased leg pain and mild headache. Both resolved	1→ brain biopsy showed neuronal ceroid lipofuscinosis Patient died 3 months later 2→ patient opted for surgery but no cortical resection could be done

Naro et al. [17]	1	Round	4 trains with 300 pulses/1 Hz with 30-second intertrain interval	Levetiracetam Valproate Lorazepam	Complete remission	6 days	None	Myoclonic jerks reappeared though less frequent and intense

Rotenberg et al. [18]	7	Figure of 8	1→ 3/1 Hz/1800 s 2→ 1/1 Hz/1600 s and 40/20 Hz/2 s followed by 1/1 Hz/1600 s 3→ 40/20 Hz/1660 s 4→ 2/1 Hz/1600 s 5→ 1/1 Hz/200 s 6→ 15/100 Hz/0.05–1.25 s & 10/1 Hz/1600–1800 s 7→ 1/1 Hz/1800 s 20/20 Hz/4 s	N/A	No effect = 2 Seizure ceased during TMS = 3 Seizure cessation after TMS = 2	Seizures ceased during TMS lasting 30 minutes 1 patient = 2 days 1 patient = >4 months	None	2/7 had no EEG response to TMS 3/7 had a short-lived response lasting 20-30 min after TMS train before relapse of clinical seizures 2/7 had lasting anticonvulsive effect throughout follow-up (2 days for 1 patient and >4 months for another)

Thordstein and Constantinescu [19]	1	Figure of 8	1/0.5 Hz/3600 s 2 days of 1/day and 6 days of 2/day	Fosphenytoin Levetiracetam Topiramate	Continuous seizures stopped, localized epileptiform activity recorded	2.5 months	None	Patient clinically improved slowly and has no epileptiform potentials 2.5 months later

Thordstein et al. [20]	2	N/A	1/0.5 Hz/3600 s daily for 2 weeks	N/A	Seizure severity decreased	N/A	None	Seizure frequency and severity both decreased

Van Haerents et al. [21]	1	N/A	3/1 Hz/600 s 11 sessions	Zonisamide Lamotrigine Phenobarbital Phenytoin	Seizure frequency progressively declined and then ceased	N/A	None	Complete seizure control and stabilization of epilepsy allowed patient to return to normal life

Wusthoff et al. [22]	1	N/A	N/A	N/A	No effect	N/A	N/A	Patient responded to ketogenic diet

Rotenberg et al. [23]	1	Figure of 8	1/1 Hz/1800 s (9 consecutive days)	Fosphenytoin Oxcarbazepine Levetiracetam Valproate Diazepam Lorazepam	Seizure suppression during treatment	Effect only during treatment	None	Patient returned to baseline seizures

rTMS: repetitive transcranial magnetic stimulation; AED: anti-epileptic drug; TMS: transcranial magnetic stimulation; AED: antiepileptic drug; N/A: not available; SE: status epilepticus; FSE: focal status epilepticus; FRSE: focal refractory status epilepticus; GRSE: generalized refractory status epilepticus; yrs: years; mons: months; h: hours; s: seconds. Rotenberg et al. [18] contains a series of patients including the case description from Rotenberg et al. [23]. Thus, the data from Rotenberg et al. [23] was not included in the final summary and analysis of data in order to avoid duplication of patient data.

**Table 3:** Oxford and GRADE level of evidence.

Reference	Study type	Oxford [29] level of evidence	GRADE [28, 30–33] level of evidence
Graff-Guerrero et al. [12]	Retrospective case series	4	D
Hyllienmark and Åmark [13]	Retrospective case report	4	D
Liu et al. [14]	Retrospective case series	4	D
Misawa et al. [15]	Retrospective case report	4	D
Morales et al. [16]	Retrospective case series	4	D
Naro et al. [17]	Retrospective case report	4	D
Rotenberg et al. [18]	Retrospective case series	4	D
Thordstein and Constantinescu [19]	Retrospective case report	4	D
Thordstein et al. [20]	Retrospective case series	4	D
Van Haerents et al. [21]	Retrospective case report	4	D
Wusthoff et al. [22]	Retrospective case report	4	D
Rotenberg et al. [23]	Retrospective case report	4	D

Rotenberg et al. [18] contains a series of patients including the case description from Rotenberg et al. [23]. Thus, the data from Rotenberg et al. [23] was not included in the final summary and analysis of data in order to avoid duplication of patient data.
