# Supplementary material for: Transcranial Magnetic Stimulation for Status Epilepticus
Source: Epilepsy Res Treat. 2015 Nov 22;2015:678074. doi: 10.1155/2015/678074 (PMC4670661; doi:10.1155/2015/678074)
Supplement: Supplementary file 1 — Appendix A displays a sequential listing of the exact search terms and operators utilized in our MEDLINE database search. This search strategy was similar for all of the databases listed. [file 678074.f1.pdf]

Appendix A: Medline Search Strategy

1. seizure.mp. [mp=ti, ab, hw, tn, ot, dm, mf, dv, kw, bt, id, cc, nm, kf, px, rx, an, ui]
2. seizure.tw.
3. epilepsy.mp. [mp=ti, ab, hw, tn, ot, dm, mf, dv, kw, bt, id, cc, nm, kf, px, rx, an, ui]
4. epilepsy.tw.
5. status epilepticus.mp. [mp=ti, ab, hw, tn, ot, dm, mf, dv, kw, bt, id, cc, nm, kf, px, rx, an, ui]
6. status epilepticus.tw.
7. electrographic seizure.mp. [mp=ti, ab, hw, tn, ot, dm, mf, dv, kw, bt, id, cc, nm, kf, px, rx, an, ui]
8. electrographic seizure.tw.
9. refractory.mp. [mp=ti, ab, hw, tn, ot, dm, mf, dv, kw, bt, id, cc, nm, kf, px, rx, an, ui]
10. refractory.tw.
11. medically refractory.mp. [mp=ti, ab, hw, tn, ot, dm, mf, dv, kw, bt, id, cc, nm, kf, px, rx, an, ui]
12. medically refractory.tw.
13. unresponsive.mp. [mp=ti, ab, hw, tn, ot, dm, mf, dv, kw, bt, id, cc, nm, kf, px, rx, an, ui]
14. unresponsive.tw.
15. no response.mp. [mp=ti, ab, hw, tn, ot, dm, mf, dv, kw, bt, id, cc, nm, kf, px, rx, an, ui]
16. no response.tw.
17. lack of response.mp. [mp=ti, ab, hw, tn, ot, dm, mf, dv, kw, bt, id, cc, nm, kf, px, rx, an, ui]
18. lack of response.tw.
19. treatment failure.mp. [mp=ti, ab, hw, tn, ot, dm, mf, dv, kw, bt, id, cc, nm, kf, px, rx, an, ui]
20. treatment failure.tw.
21. TMS.mp. [mp=ti, ab, hw, tn, ot, dm, mf, dv, kw, bt, id, cc, nm, kf, px, rx, an, ui]
22. TMS.tw.

23. transcranial magnetic stimulation.mp. [mp=ti, ab, hw, tn, ot, dm, mf, dv, kw, bt, id, cc, nm, kf, px, rx, an, ui]

24. transcranial magnetic stimulation.tw.

25. transcranial magnetic.mp. [mp=ti, ab, hw, tn, ot, dm, mf, dv, kw, bt, id, cc, nm, kf, px, rx, an, ui]

26. transcranial magnetic.tw.

27. transcranial stimulation.mp. [mp=ti, ab, hw, tn, ot, dm, mf, dv, kw, bt, id, cc, nm, kf, px, rx, an, ui]

28. transcranial stimulation.tw.

29. magnetic stimulation.mp. [mp=ti, ab, hw, tn, ot, dm, mf, dv, kw, bt, id, cc, nm, kf, px, rx, an, ui]

30. magnetic stimulation.tw.

31. magnet therapy.mp. [mp=ti, ab, hw, tn, ot, dm, mf, dv, kw, bt, id, cc, nm, kf, px, rx, an, ui]

32. magnet therapy.tw.

33. cortical modulation.mp. [mp=ti, ab, hw, tn, ot, dm, mf, dv, kw, bt, id, cc, nm, kf, px, rx, an, ui]

34. cortical modulation.tw.

35. 1 or 2 or 3 or 4 or 5 or 6 or 7 or 8

36. 9 or 10 or 11 or 12 or 13 or 14 or 15 or 16 or 17 or 18 or 19 or 20

37. 21 or 22 or 23 or 24 or 25 or 26 or 27 or 28 or 29 or 30 or 31 or 32 or 33 or 34

38. 35 and 36

39. 37 and 38

40. remove duplicates from 39
